# Supplementary material for: Nanoscale design of polarization in ultrathin ferroelectric heterostructures
Source: Nat Commun. 2017 Nov 10;8:1419. doi: 10.1038/s41467-017-01620-2 (PMC5681682; doi:10.1038/s41467-017-01620-2)
Supplement: Supplementary file 1 — Supplementary Information [file 41467_2017_1620_MOESM1_ESM.pdf]

Supplementary Information.

Supplementary Figures

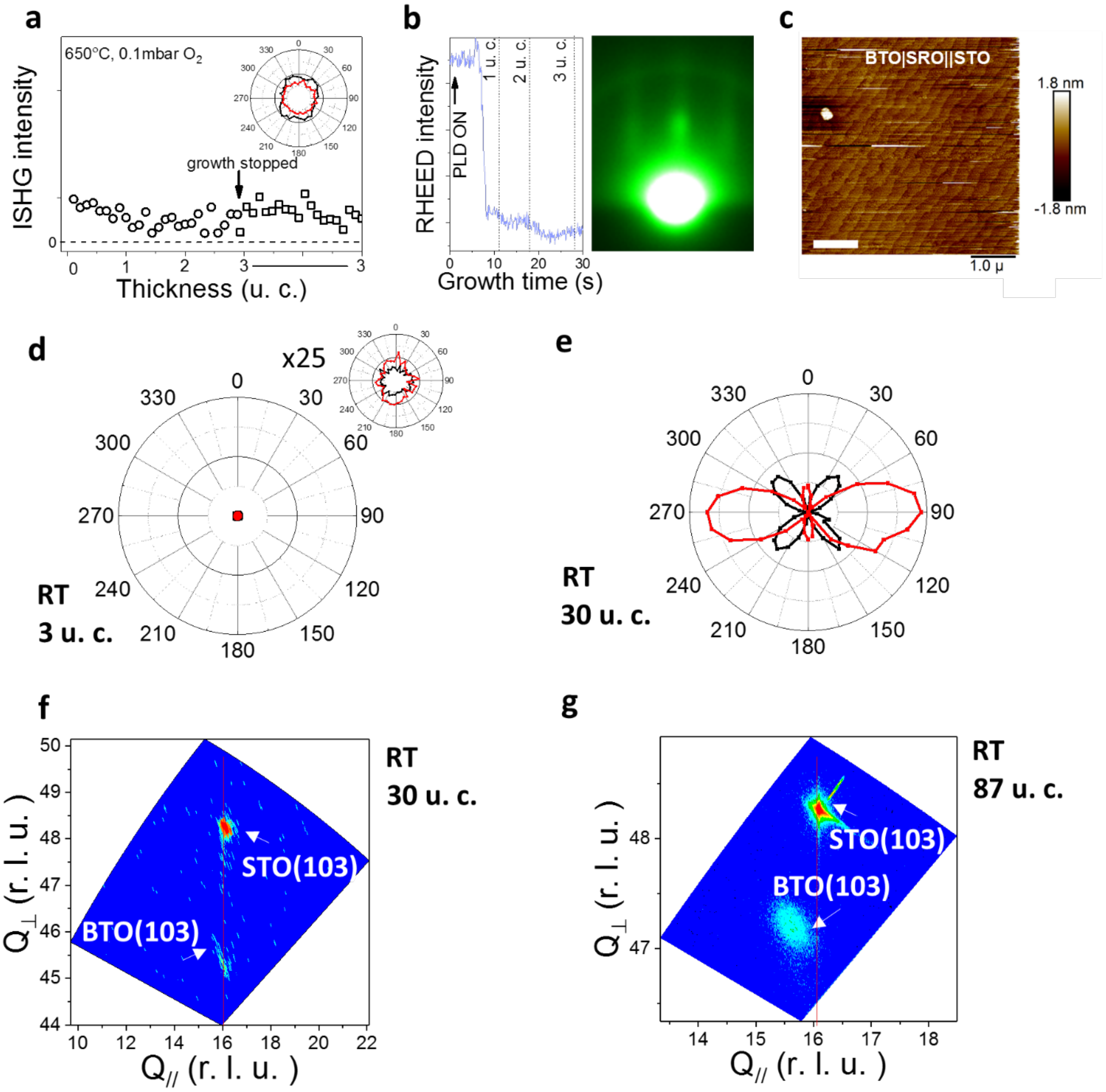

Supplementary Figure 1

Change of ferroelectric critical thickness with temperature for the $BaTiO_3|SrRuO_3||SrTiO_3$ films of Fig. 1. (a) In-situ second harmonic generation (ISHG) signal during growth of a $BaTiO_3$ (BTO) film of 3 unit cells (u. c.). Only an unpolarized background signal is observed. This signal does not change with the ongoing deposition of the BTO up to the third unit cell or after this deposition has been stopped at three unit cells coverage. The inset shows the polarization dependence of the ISHG yield (varying the polarization of the incident fundamental light and measuring a fixed polarization component of the emitted ISHG light). **(b)** In situ reflection high energy electron diffraction (RHEED) monitoring during growth. The RHEED pattern is shown in the right panel. **(c)** The room-temperature (RT) topography of the film from (a) reveals homogeneous growth. The scale bar corresponds to 1 μm. **(d, e)** Polarization dependence of the ISHG yield at room temperature for films of 3 and 30 u. c., the comparison of (d) to the data taken at the growth temperature and to the room-temperature data taken on the film of 30 u. c. reveals no detectable

spontaneous room-temperature polarization in the film of 3 u. c.: The critical thickness measured at the growth temperature is also valid at room temperature. This result was confirmed by piezoresponse force microscopy (PFM) measurements performed after tip-voltage poling. **(f, g)** Reciprocal space mapping of the $SrTiO_3$ and BTO (103) reflections indicating a strain state for a 30 u. c. thick BTO film and an in-plane strain relaxation for an 87 u. c. thick BTO film, respectively. This indicates that a 3 u. c. thick film is fully strained to the substrate lattice.

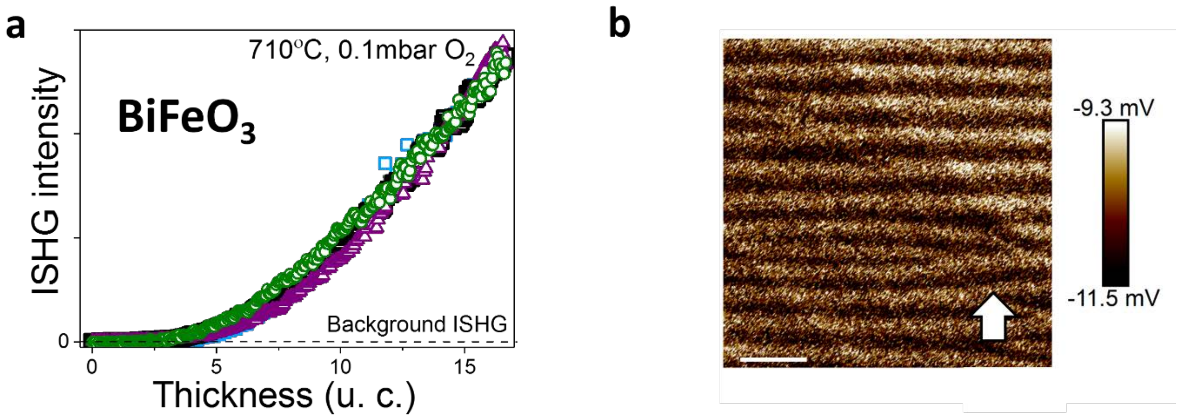

Supplementary Figure 2

Reproducibility and domain structure in the growth of the $BiFeO_3|SrRuO_3||DyScO_3$ films of Fig. 2. (a) In-situ second harmonic generation (ISHG) signal from 4 different experimental runs under the same growth conditions. Because of small variations in the path of the in- and outgoing light at ω and 2ω, respectively, data were normalized by setting the maximum ISHG yield to 1. Within the statistical noise, the emergence of the polarization is the same in all runs. **(b)** In-plane piezoresponse force microscopy scan on one of the samples in (a) reveals a ferroelectric $BiFeO_3$ stripe-domain pattern. The net in-plane polarization direction is indicated by the arrow. The scale bar corresponds to 1 μm.

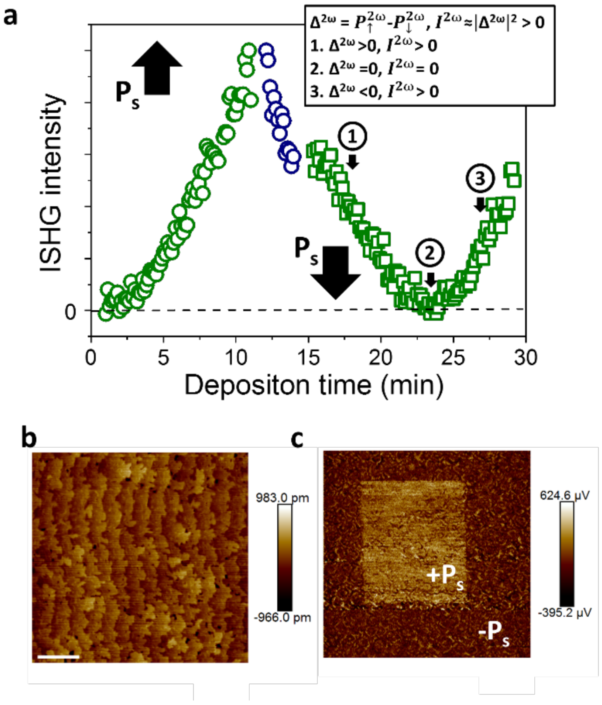

Supplementary Figure 3

In-situ second harmonic generation (ISHG) interference effects between oppositely polarized $BiFeO_3$ (BFO) films. (a) Continuation of the deposition of down-polarized BFO onto the heterostructure shown in Fig. 4e. Comparison of the ISHG yield at points ①, ②, ③ shows that three cases have to be distinguished in the interference of the ISHG waves from the up- and down-polarized BFO layers. The amplitudes of the respective waves are denoted as $P_\uparrow^{2\omega}$ and $P_\downarrow^{2\omega}$. As explained in the main text, these waves possess a 180° phase difference. Although the net second harmonic generation wave experiences a change of sign when going from ① to ③, the ISHG *intensity* is proportional to $|P^{2\omega}|^2$ and thus always positive. This leads to the renewed increase of the ISHG yield once it has passed zero in ②. **(b)** Topography of the heterostructure from (a). The scale bar corresponds to 1 µm. **(c)** Out-of-plane piezoresponse force microscopy (PFM) scan of the heterostructure from (a) with dark and bright areas representing up- and down-polarized regions, respectively. As expected from the ISHG measurement in (a) the top BFO layer is down-polarized and undergoes polarization reversal after tip poling at 4 V in the quadratic center region. Note that the bottom BFO layer is inaccessible to the PFM scan. Its orientation is revealed by ISHG only.

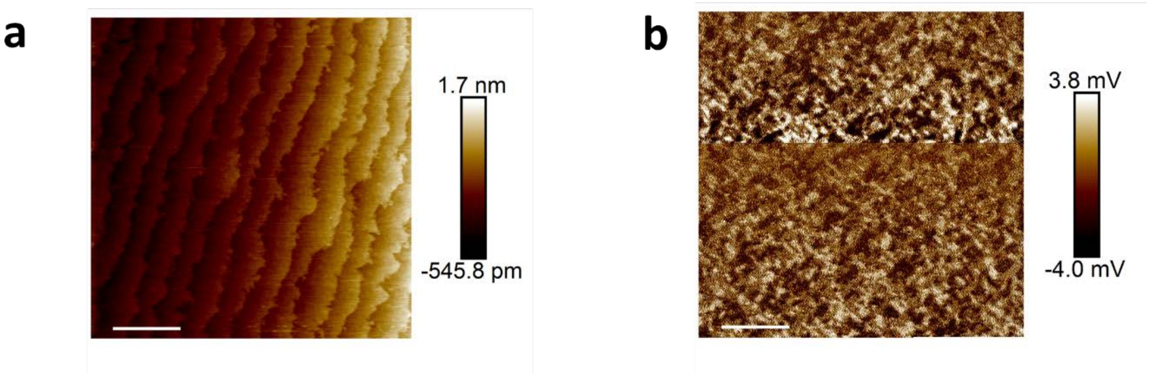

Supplementary Figure 4

Topography and in-plane piezoresponse force microscopy (PFM) scan of a $(BiFeO_3|SrRuO_3)_n||DyScO_3$ sample as in Fig. 3. We have n = 5, $BiFeO_3$: 20 unit cells, $SrRuO_3$: 10 unit cells. **(a)** Topography. Homogeneous growth is maintained until completion of the deposition. **(b)** In-plane PFM scan. As expected and explained in the main text, the film exhibits a ferroelectric multi-domain structure[1]. The scale bars correspond to 1 µm.

Supplementary references

1 Trassin, M., De Luca, G., Manz, S. & Fiebig, M. Probing ferroelectric domain engineering in $BiFeO_3$ thin films by second harmonic generation. *Adv. Mater*. **27,** 4871–4876 (2015).
